# CT imaging-based histogram features for prediction of EGFR mutation status of bone metastases in patients with primary lung adenocarcinoma

**DOI:** 10.1186/s40644-019-0221-9

**Published:** 2019-06-07

**Authors:** Tong-xu Shen, Lin Liu, Wen-hui Li, Ping Fu, Kai Xu, Yu-qing Jiang, Feng Pan, Yan Guo, Meng-chao Zhang

**Affiliations:** 1 Department of Radiology, China-Japan Union Hospital of Jilin University, NO.126 Xiantai Street, Changchun, 130033 Jilin China; 2 College of Computer Science and Technology, Jilin University, NO.2699 Qianjin Street, Changchun, 130012 Jilin China; 3 Department of Ultrasound, China-Japan Union Hospital of Jilin University, NO.126 Xiantai Street, Changchun, 130033 Jilin China; 4 GE Healthcare, China, NO.69 Heping North Street, Shenyang, 110000 Liaoning China

**Keywords:** Bone neoplasms, Metastasis, Histogram analysis, Lung adenocarcinoma, Epidermal growth factor receptor

## Abstract

**Objective:**

To identify imaging markers that reflect the epidermal growth factor receptor (EGFR) mutation status by comparing computed tomography (CT) imaging-based histogram features between bone metastases with and without EGFR mutation in patients with primary lung adenocarcinoma.

**Materials and methods:**

This retrospective study included 57 patients, with pathologically confirmed bone metastasis of primary lung adenocarcinoma. EGFR mutation status of bone metastases was confirmed by gene detection. The CT imaging of the metastatic bone lesions which were obtained between June 2014 and December 2017 were collected and analyzed. A total of 42 CT imaging-based histogram features were automatically extracted. Feature selection was conducted using Student’s t-test, Mann-Whitney U test, single-factor logistic regression analysis and Spearman correlation analysis. A receiver operating characteristic (ROC) curve was plotted to compare the effectiveness of features in distinguishing between EGFR(+) and EGFR(−) groups. DeLong’s test was used to analyze the differences between the area under the curve (AUC) values.

**Results:**

Three histogram features, namely range, skewness, and quantile 0.975 were significantly associated with EGFR mutation status. After combining these three features and combining range and skewness, we obtained the same AUC values, sensitivity and specificity. Meanwhile, the highest AUC value was achieved (AUC 0.783), which also had a higher sensitivity (0.708) and specificity (0.788). The differences between AUC values of the three features and their various combinations were statistically insignificant.

**Conclusion:**

CT imaging-based histogram features of bone metastases with and without EGFR mutation in patients with primary lung adenocarcinoma were identified, and they may contribute to diagnosis and prediction of EGFR mutation status.

**Electronic supplementary material:**

The online version of this article (10.1186/s40644-019-0221-9) contains supplementary material, which is available to authorized users.

## Introduction

In recent years, molecular targeted therapy has been widely accepted for lung adenocarcinoma, and the epidermal growth factor receptor (EGFR) gene is a vital target of lung adenocarcinoma. The selection of epidermal growth factor receptor tyrosine kinase inhibitors (EGFR-TKIs) for treatment when EGFR mutation is positive can prolong progression-free survival [1, 2]. In the progression of lung adenocarcinoma, synchronous and metachronous bone metastases are a more common phenomenon, with a prevalence of 30–40% [3]. Recent studies have shown a high degree of consistency in EGFR mutation status between primary pulmonary lesions and metastatic bone lesions [4]. Thus, when the specimens of primary lesions are not available in patients with advanced lung adenocarcinoma, the EGFR mutation status of metastatic lesions can be analyzed to represent the primary lesions for guiding the treatment. Typically, tissue specimens for EGFR mutation detection are obtained by biopsy. However, the results of mutation detection in biopsy samples are not accurate enough, because when the proportion of cancerous cells in the sample is low, the mutation ratios in metastatic lesions are reduced and occasionally undetectable [5]. Also, biopsy is associated with some complications such as hematoma, and important vessel and nerve injuries [6]. In addition, when EGFR mutation is positive, synchronous metastatic lesions may be enlarged in size or may even increase in number in the patients receiving EGFR-TKIs treatment [5]. Although this is a minor phenomenon, it indicates that there may be heterogeneity between the primary tumors and metastases. Moreover, there are frequent changes in the gene mutation status of tumors in metachronous metastases, such as breast cancer [7, 8]. Although whether this change existed is still uncertain in the metachronous bone metastases of lung adenocarcinoma, it suggests that we should pay attention to the possibility of inconsistency. Therefore, we need a non-invasive and accurate method to distinguish EGFR mutation status of the metastatic lesions throughout the treatment process of lung adenocarcinoma.

Recently, a number of studies have demonstrated that radiomics features were significantly correlated with potential gene expression patterns and they might provide additional assistance for individualized treatment and efficacy monitoring [9–11]. Studies have shown that the use of first-order histogram features in radiomics features based on computed tomography (CT) could predict EGFR mutation status in non-small cell lung cancer (NSCLC) [12]. However, the correlation between the CT histogram features of bone metastases and their EGFR mutation status suggesting that the CT histogram features are the biomarkers of EGFR has not been well demonstrated.

Therefore, the purpose of our study was to identify the imaging markers that reflect the EGFR mutation status by comparing CT imaging-based histogram features between bone metastases with and without EGFR mutation in patients with primary lung adenocarcinoma.

## Materials and methods

### Patients

**Fig. 1:**
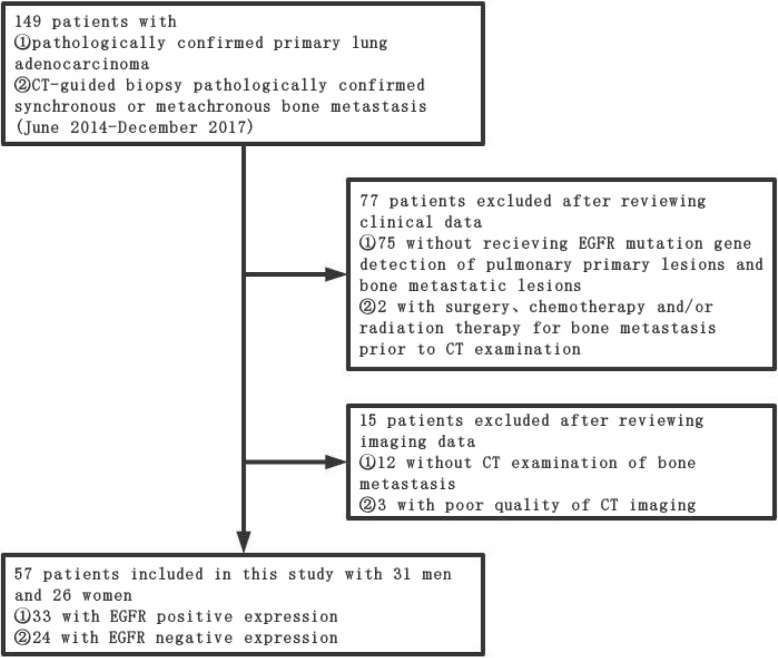
Flowchart for selecting patients

This retrospective study was approved by our institutional review board, and the requirement for obtaining informed consent was waived. Clinical and imaging information of all patients were obtained through medical record system and follow-up. We collected 149 patients with pathologically confirmed primary lung adenocarcinoma who were confirmed to have synchronous or metachronous bone metastasis by CT-guided biopsy pathological examination from June 2014 to December 2017. By reviewing the clinical and imaging data of these patients, we excluded the patients who had not received the test for EGFR mutation status in primary pulmonary lesions and metastatic bone lesions (*N* = 75); who had not undergone CT examination (*N* = 12); and who had undergone surgery, chemotherapy, and radiation therapy for bone metastases before CT examination (*N* = 2). In addition, patients with poor CT image quality (*N* = 3) were also excluded. After the above-mentioned screening, 57 patients were finally included in the study (Fig. 1). We also collected data on the clinical characteristics of each patient, including age at diagnosis, gender, and smoking status.

### EGFR mutation analysis

DNA was extracted from formalin-fixed paraffin-embedded (FFPE) tumor sections using the QIAamp DNA FFPE Tissue Kit (Qiagen). Mutations of EGFR (exons 18, 19, 20 and 21) were analyzed by fluorescent quantitative polymerase chain reaction (PCR) method.

### CT protocols

All of the examinations were performed with GE MEDICAL SYSTEMS Discovery CT750 HD BASE (M) 64-row multidetector CT scanner without contrast medium. Scanning parameters were as follows: tube voltage, 120 kV; automatic tube current adjustment technology; standard soft-tissue algorithm reconstruction; scanning thickness, 1.25 mm; reconstruction interval, 1.25 mm; rotation speed, 0.6 s/turn; and matrix, 512*512.

### Data preprocessing

**Fig. 2:**
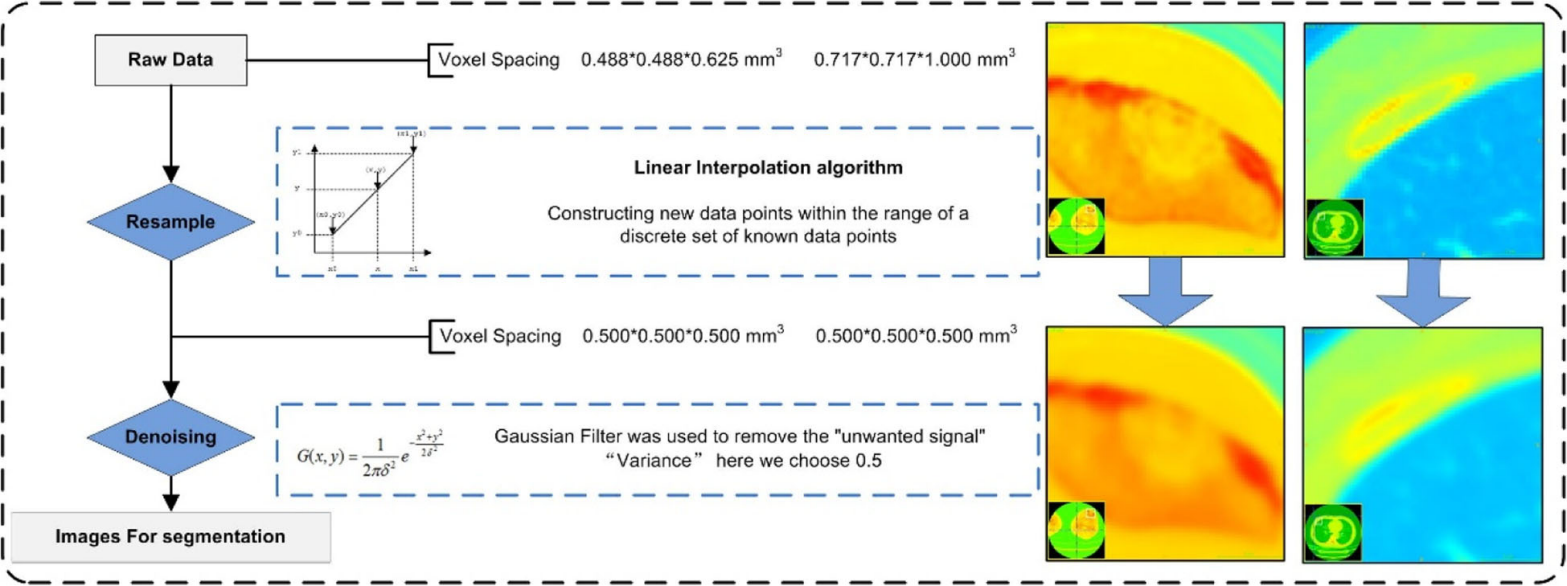
Flowchart for data preprocessing. We selected a left knee CT and a chest CT, and correspondingly the sites of tumor segmentation were located in the patella and rib, respectively. The voxel spacing of left knee CT and chest CT were 0.488*0.488*0.625 mm^3^ and 0.717*0.717*1.000 mm^3^. By resampling, their voxel spacing were both 0.500*0.500*0.500 mm^3^. Then denoising was used to get the images for segmentation

All of the CT images obtained from these 57 patients were plain CT scan images, including 1 case with CT of the cervical vertebrae, 1 case with CT of the thoracic vertebrae CT, 1 case with CT of the left knee, 2 cases with CT of the lumbar vertebrae, and the remaining 52 cases with chest CT. The image quality and image noise were different in different parts of the body, and their raw data had different voxel spacing. Therefore, data preprocessing was necessary to ensure that the imaging features were calculated using the same specifications [13]. First, all of the raw data were resampled to a common voxel spacing of 0.500*0.500*0.500 mm^3^ by using linear interpolation algorithm to construct new data points within the range of a discrete set of known data points. Then for denoising, the Gaussian Filter was used to remove the “unwanted signal”. “Variance” here we chose 0.5. Finally, the images after data preprocessing were used for tumor segmentation. We selected CT images of bone metastases from two different anatomical sites as examples to show the data preprocessing (Fig. 2).

### Tumor segmentation

Complete thin-layer CT images after data preprocessing were stored in Digital Imaging and Communications in Medicine (DICOM) format and uploaded to ITK-SNAP software (http://www.itk-snap.org/) for three-dimensional (3D) manual segmentation of the region of interest (ROI) [14] with a window width of 1500 HU and a window level of 400 HU. The largest bony metastatic lesion on CT was chosen as ROI. The whole tumor was manually segmented by a radiologist who did not have any knowledge about the clinical information of patients, including the cervical vertebrae, thoracic vertebrae, lumbar vertebrae, clavicle, sternum, scapula, ribs, and patella, and then the segmentation was checked by a senior radiologist.

### Feature extraction and selection

The above images and ROIs were imported into A.K. software (Artificial Intelligence Kit, A.K., GE Healthcare, China), and 42 histogram features based on the individual pixel values of CT images were automatically extracted.

**Fig. 3:**
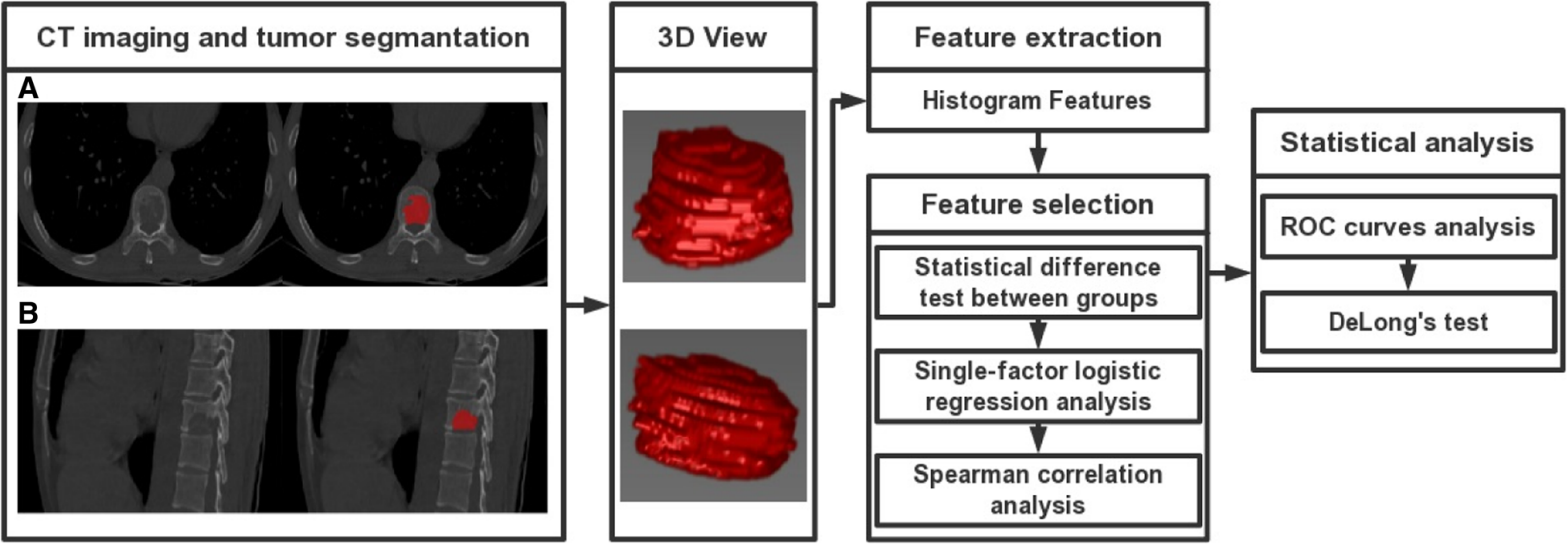
Flowchart illustrating the histogram analysis in the study. **a** and **b** are axial and sagittal positions of original CT images obtained from a case with bone metastases of lung adenocarcinoma. 3D ROIs were segmented manually and reconstructed, as shown in the figure. Histogram features were generated automatically and selected by using the “MRMR” method

Minimum redundancy maximum relevancy (MRMR) feature selection [15] was implemented to select the optimal features, which maximally distinguished between EGFR positive expression and EGFR negative expression while minimizing intra-feature correlation among these 42 features. Here, we performed the following two steps: In the first step, Shapiro-Wilk test was used to test for normality of the features in each group. The features of normal distribution were tested for homogeneity of variance by using Bartlett’s test. Then the features with homogeneity of variance were analyzed by Student’s t-test, and the other features were analyzed by Mann-Whitney U test. All of the features that were significantly different between the two groups were substituted into single-factor logistic regression analysis to determine the features that were maximally relevant to the EGFR mutation status. A significance level of 0.05 was set as the threshold. In the second step, Spearman correlation analysis was performed to eliminate redundancy. A correlation coefficient R > 0.9 was selected as the cutoff for strong relationships, in which one of the two features was excluded to minimize intra-feature correlation (Fig. 3). The feature selection process was completed by using R Studio (Version 1.0.143–© 2009–2016 R Studio, Inc.).

Nine morphological features of the bony metastatic lesion were also automatically extracted to show the correlation with histogram features, including sphericity, surface area, volume CC, volume MM, surface volume ratio, maximum 3D diameter, compactness1, compactness2, and spherical disproportion.

### Statistical analysis

Clinical characteristics of the two groups were compared using Student’s t-test and chi-square test, and *p* < 0.05 indicated a significant difference. The receiver operating characteristic (ROC) curve was constructed to assess the discriminative performance of the histogram features. The area under the curve (AUC), specificity, and sensitivity were calculated, and the differences between the AUC values were analyzed by DeLong’s test. All of the statistical analyses were performed using SPSS 22.0 for Windows.

## Results

### Patient demographic characteristics

Fifty-seven patients were divided into two groups based on the results of EGFR mutation status of metastatic bone lesions; 33 patients showed EGFR positive expression and 24 patients showed EGFR negative expression. On the basis of their primary pulmonary lesions, 32 patients showed EGFR positive expression and 25 patients showed EGFR negative expression. The clinical characteristics of the two groups are shown in Table 1. There was no statistically significant difference between EGFR mutation status and gender or age (*p* ≥ 0.05). There was a statistically significant difference between EGFR mutation status and smoking status. The EGFR-positive group had more non-smokers than the EGFR-negative group.

**Table 1:** Characteristics of patients in the EGFR(+) group and EGFR(−) group

Clinical data		EGFR(+) (*N* = 33)	EGFR(−) (*N* = 24)	*P* value
Age /Mean ± SD		59.00 ± 9.53	60.36 ± 7.03	0.104
Gender /No. (percentage)	Male	15(45.45%)	16(66.67%)	0.112
	Female	18(54.55%)	8(33.33%)	
Smoking status/No. (percentage)	(+)^#^	6(18.18%)	15(62.50%)	0.001^*^
	(−) ^#^	27(81.82%)	9(37.50%)	

Footnotes: (1) N, number

(2) * significant difference (p < 0.05) between the two groups

(3) (+)# smoked, while (−)# never smoked

### Feature selection

**Fig. 4:**
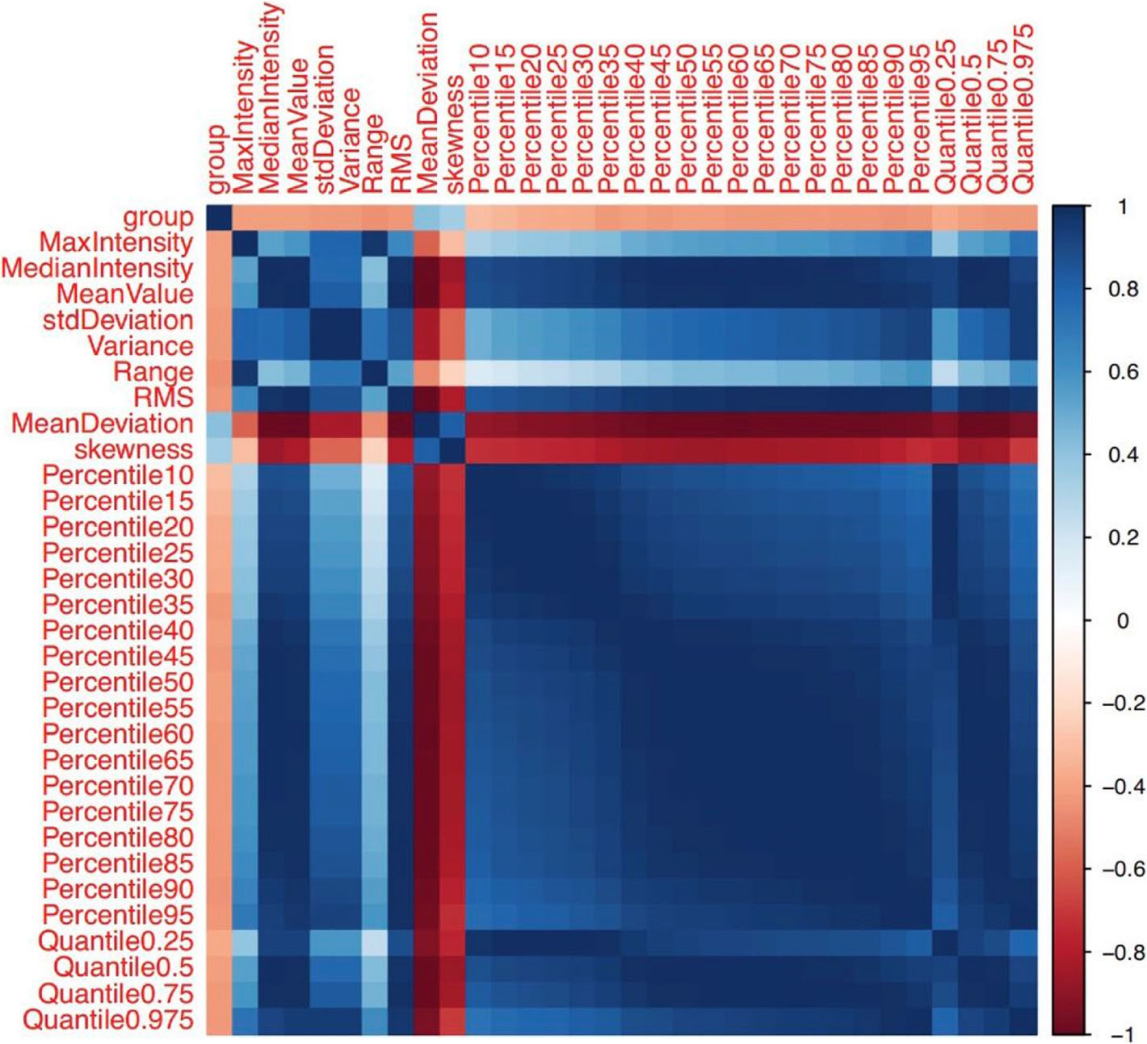
The correlation heat map. Thirty-one features were maximally relevant to the EGFR status based on the first selection step. Spearman correlation coefficient matrix, used to eliminate redundancy in the second step, is shown in the heat map. For the color scale, dark blue indicates a positive correlation, while dark red indicates a negative correlation. The deeper the color, the stronger the relationship. “Group” indicates the EGFR status confirmed by gene detection. |R| > 0.9 was considered to indicate a strong relationship with each other, in which one of the two features was eliminated. Finally, range, skewness, and quantile 0.975 remained the representative features

**Fig. 5:**
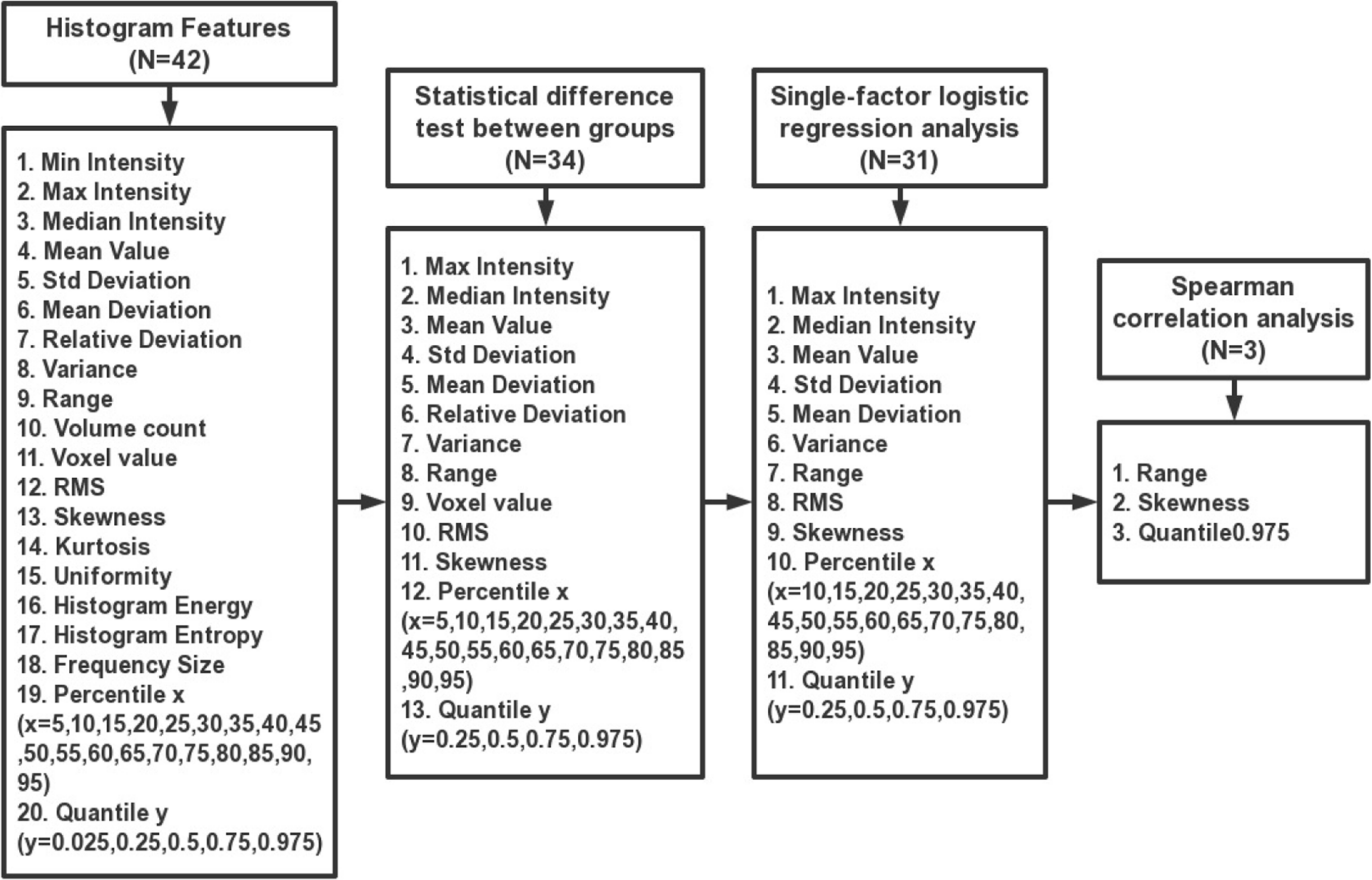
Details of the representative histogram feature selection

**Fig. 6:**
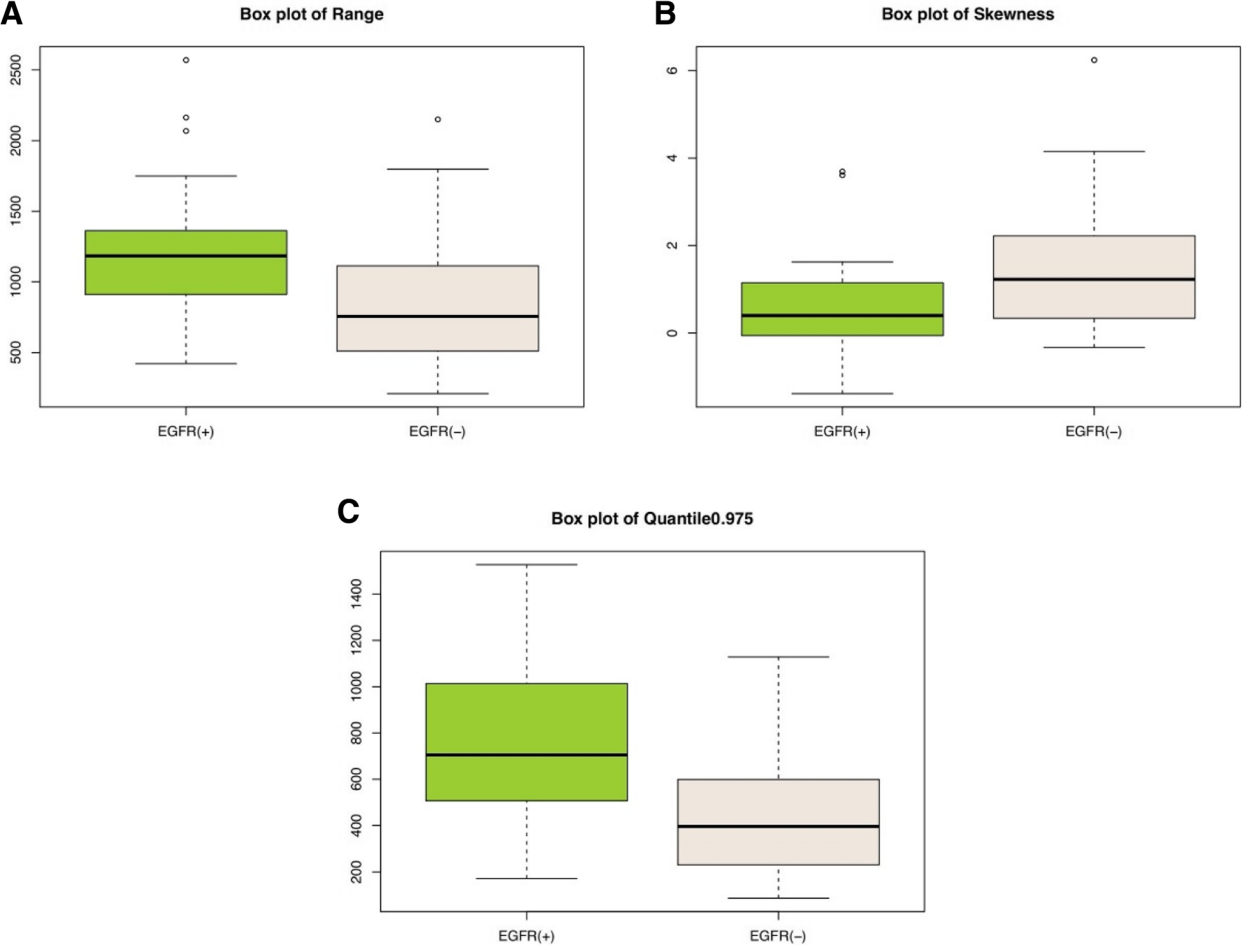
Box plots show the relationship of CT imaging-based histogram features such as range (**a**), skewness (**b**) and quantile 0.975 (**c**) with the EGFR mutation status

From the 42 features listed in Table 2, we selected 34 features that had a potential predictive ability on the basis of a statistically significant difference between the two groups. Then we entered these features one by one into the single-factor logistic regression test, and 31 features were found to be statistically significant (*p* < 0.05). After using Spearman correlation analysis to remove redundancy, which is shown in the form of a heat map in Fig. 4, three features, namely, range (*p* = 0.001), skewness (*p* = 0.011), and quantile 0.975 (*p* = 0.001), were the most representative and were significantly correlated with the EGFR mutation status. The meanings of the three independent features are as follows: Range refers to the range of voxel intensity values of the tumor, namely maximum-minimum; skewness refers to the degree of asymmetry in the distribution of pixel intensity values within the tumor; and quantile 0.975 refers to the numerical point that divides the probability distribution range of a random variable into 0.975 equal parts. The details of the representative histogram feature selection and the results of the most representative features are shown in Figs. 5 and 6. Figure 6 shows that the value of range in the EGFR(+) group was 1183.00 (888.00, 1401.00); and it was significantly higher than that in the EGFR(−) group, which showed a value of 756.5 (478.25, 1113.25). The value of skewness in the EGFR(+) group was 0.40 (− 0.19, 1.17), and it was significantly lower than that in the EGFR(−) group, which showed a value of 1.22 (0.32, 2.32). The value of quantile 0.975 was 704.95 (488.12, 1030.14) in the EGFR(+) group, and it was significantly higher than that in the EGFR(−) group, which showed a value of 395.74 (229.79, 606.97).

**Table 2:** Forty-two histogram features in the EGFR(+) group and the EGFR(−) group are presented as median (25, 75%), respectively, and a *p* value was derived on the basis of a statistically significant difference between each feature and EGFR mutation status

Features	EGFR(+)	EGFR(−)	*P* value
Min Intensity	−58.00 (− 212.00, 2.00)	−16.00 (−67.00, 26.75)	0.106
Max Intensity	1073.00 (894.50, 1272.00)	772.50 (449.50, 1077.50)	0.002^*^
Median Intensity	369.35 (106.54, 565.63)	108.92 (70.63, 208.23)	0.002^*^
Mean Value	373.53 (140.51, 551.41)	131.17 (84.15, 227.16)	0.002^*^
Std Deviation	145.44 (118.97, 245.85)	88.09 (57.75, 155.43)	0.001^*^
Variance	21,151.80 (14,163.10, 60,442.60)	7766.17 (3340.58, 24,158.58)	0.001^*^
Volume Count	6047.00 (2462.00, 14,622.50)	4164.00 (1623.75, 9188.50)	0.245
Voxel Value Sum	1,840,000.00 (492,161.00, 7,150,000.00)	745,705.00 (199,724.75, 2,115,000.00)	0.013^*^
Range	1183.00 (888.00, 1401.00)	756.50 (478.25, 1113.25)	0.001^*^
RMS	389.84 (196.88, 594.39)	183.50 (100.74, 294.90)	0.001^*^
Mean Deviation	−118.53 (− 296.44, 114.50)	123.84 (27.84, 170.85)	0.002^*^
Relative Deviation	− 1058.43 (− 5577.42, 2235.28)	2337.26 (− 404.51, 8608.66)	0.009^*^
Skewness	0.40 (−0.19, 1.17)	1.22 (0.32, 2.32)	0.011^*^
Kurtosis	0.62 (−0.39, 2.18)	2.16 (−0.14, 7.21)	0.165
Uniformity	0.52 (0.24, 0.66)	0.32 (0.19, 0.63)	0.225
Histogram Energy	0.01 (0.01, 0.01)	0.01 (0.01, 0.02)	0.213
Histogram Entropy	7.01 (6.72, 7.32)	6.76 (6.22, 7.24)	0.137
Frequency Size	6046.00 (2461.00, 14,621.50)	4163.00 (1622.75, 9187.50)	0.245
Percentile 5	111.25 (30.33, 195.40)	34.94 (6.94, 104.10)	0.047^*^
Percentile 10	187.31 (41.50, 283.71)	40.98 (21.68, 125.22)	0.021^*^
Percentile 15	220.21 (49.21, 337.86)	45.55 (31.17, 139.97)	0.012^*^
Percentile 20	239.05 (56.38, 384.60)	49.92 (37.47, 151.24)	0.007^*^
Percentile 25	267.93 (62.89, 433.14)	53.39 (41.39, 160.71)	0.005^*^
Percentile 30	298.39 (68.42, 468.12)	56.89 (47.82, 170.64)	0.004^*^
Percentile 35	316.22 (75.99, 493.25)	60.53 (52.20, 179.06)	0.001^*^
Percentile 40	333.97 (82.54, 517.86)	70.52 (62.42, 187.81)	0.002^*^
Percentile 45	349.95 (93.92, 541.96)	91.71 (66.74, 198.01)	0.002^*^
Percentile 50	368.77 (107.44, 564.66)	109.48 (70.20, 208.48)	0.002^*^
Percentile 55	382.74 (123.39, 583.52)	119.17 (74.45, 221.36)	0.002^*^
Percentile 60	395.80 (140.10, 608.67)	130.12 (79.11, 240.573)	0.001^*^
Percentile 65	409.11 (161.26, 638.45)	140.19 (84.52, 265.378)	0.001^*^
Percentile 70	430.82 (183.03, 673.18)	153.89 (93.23, 296.70)	0.001^*^
Percentile 75	452.70 (207.13, 724.34)	168.34 (101.78, 333.79)	0.001^*^
Percentile 80	469.42 (235.15, 777.98)	186.77 (117.55, 376.72)	0.001^*^
Percentile 85	504.88 (270.93, 833.95)	211.65 (132.75, 433.35)	0.001^*^
Percentile 90	558.05 (320.99, 880.90)	272.33 (150.66, 498.35)	0.001^*^
Percentile 95	638.65 (403.52, 970.84)	331.95 (184.23, 534.20)	0.001^*^
Quantile 0.025	83.39 (7.50, 139.66)	27.60 (0.02, 83.78)	0.272
Quantile 0.25	267.93 (62.89, 433.14)	53.39 (41.39, 160.71)	0.005^*^
Quantile 0.5	368.77 (107.44, 564.66)	109.48 (70.20, 208.48)	0.002^*^
Quantile 0.75	452.70 (207.13, 724.34)	168.34 (101.78, 333.79)	0.001^*^
Quantile 0.975	704.95 (488.12, 1030.14)	395.74 (229.79, 606.97)	0.001^*^

Footnotes: (1) * significant difference (*p* < 0.05) between the two groups

(2) Abbreviations: RMS, root mean square; Std, standard

**Fig. 7:**
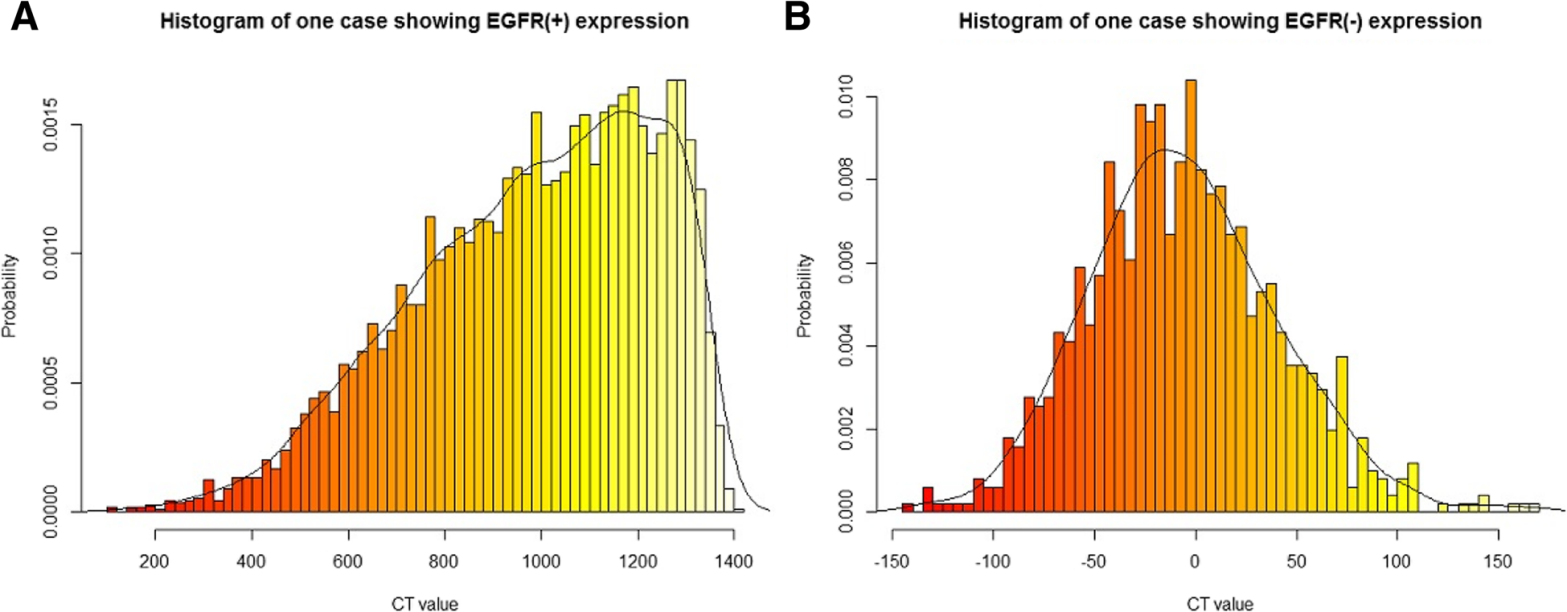
Representative histograms based on CT images. In the figure, the horizontal axis represents CT value, and the vertical axis represents the probability of which the corresponding CT value appears. **a** The histogram of a case showing EGFR positive expression; a 59-year-old woman diagnosed with primary lung adenocarcinoma that was confirmed as synchronous bone metastasis. The value of range was 1301.00. **b** The histogram of a case showing EGFR negative expression; a 67-year-old man diagnosed with primary lung adenocarcinoma that was confirmed as synchronous bone metastasis. The value of range was 312.00

We also randomly selected a single respective case from the EGFR(+) group and the EGFR(−) group as an example and created the histogram shown in Fig. 7. As seen in the figure, the value of range in the EGFR-positive patient was significantly higher than that in the EGFR-negative patient, while the value of skewness in the EGFR-positive patient was lower than that in the EGFR-negative patient. This finding was consistent with the above results.

We used the form of a heat map to elaborate the correlation between the three histogram features identified and the morphological features, as shown in the Additional file 1: Figure S1. There was no statistically significant correlation between the three histogram features and the morphological features, and there was no statistically significant correlation between EGFR expression and the morphological features. Finally, these nine morphological features were excluded.

### ROC curve analysis

**Fig. 8:**
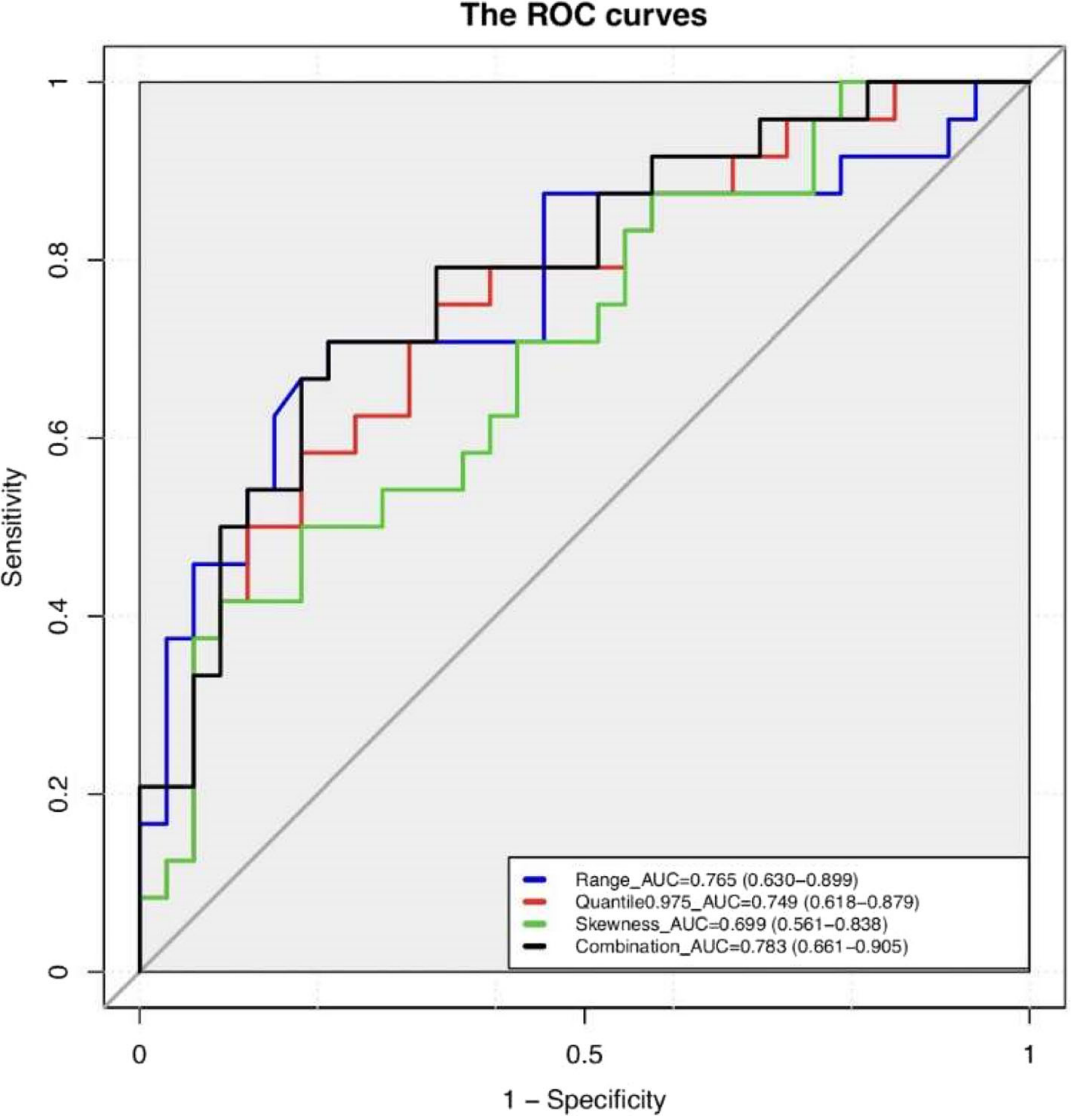
ROC curves and AUC values for the three features, and the ROC curve for the combination of the three features, including range (blue line), quantile 0.975 (red line), skewness (green line), and for the combination of the three features (black line)

We constructed ROC curves for these three features and for the combination of these three features. The results of ROC curve analysis are shown in Fig. 8. The AUC value of range was 0.765 (95% confidence interval [CI]: 0.630–0.899), the AUC value of skewness was 0.699 (95% CI: 0.561–0.838), and the AUC value of quantile 0.975 was 0.749 (95% CI: 0.618–0.879). When these three features were combined, the AUC value increased to 0.783 (95% CI: 0.661–0.905). With respect to the combination of these three features, the highest AUC value was achieved. In addition, sensitivity and specificity were calculated to evaluate the performance of the values of these features (Table 3). The sensitivity and specificity of these three features and the sensitivity and specificity of their combination were 0.788 and 0.708, 0.417 and 0.909, 0.667 and 0.750, and 0.708 and 0.788, respectively. Furthermore, we also calculated the AUC, sensitivity and specificity of the combination of every two features, which were also shown in Table 3. The AUC values of range and skewness, range and quantile 0.975, and skewness and quantile 0.975 were 0.783 (95% CI: 0.661–0.905), 0.769 (95% CI: 0.641–0.897), and 0.750 (95% CI: 0.618–0.882), respectively. The sensitivity and specificity were 0.708 and 0.788, 0.625 and 0.848, and 0.667 and 0.788, respectively. Compared with the combination of the three features, the combination of range and skewness had the same AUC value, sensitivity and specificity. Finally, the differences between the AUC values in the seven groups were analyzed in pairs, and they were not statistically significant, as shown in the Additional file 1: Table S1.

**Table 3:** Results of ROC curve analysis

Feature		Range	Skewness	Quantile 0.975	Combination (R + S)	Combination (R + Q)	Combination (S + Q)	Combination (R + S + Q)
AUC		0.765	0.699	0.749	0.783	0.769	0.750	0.783
Threshold		0.501	0.496	0.417	0.460	0.531	0.519	0.462
Specificity		0.708	0.909	0.750	0.788	0.848	0.788	0.788
Sensitivity		0.788	0.417	0.667	0.708	0.625	0.667	0.708
95% CI	Lower bound	0.630	0.561	0.618	0.661	0.641	0.618	0.661
	Upper bound	0.899	0.838	0.879	0.905	0.897	0.882	0.905

Footnote: R, range; S, skewness; Q, quantile 0.975

## Discussion

In this study, we analyzed CT imaging-based histogram features of bone metastases with and without EGFR mutation in patients with primary lung adenocarcinoma. We not only calculated AUC, sensitivity and specificity of the three independent features extracted and various combinations of these features, but also analyzed the differences between the AUC values. Our results indicated that histogram features may be helpful to diagnose and predict EGFR mutation status of metastatic bone lesions in patients with primary lung adenocarcinoma.

We finally obtained the following three independent features: range, skewness, and quantile 0.975. Range approximately describes the degree of variation of pixel intensity in the tumor and is related to intratumoral non-uniformity levels [16]. The results of our study showed that compared with bone metastases without EGFR mutation, bone metastases with EGFR mutation tended to have a higher range, i.e., the levels of intratumoral non-uniformity were higher. The intratumoral non-uniformity was similar to tumor heterogeneity. Previous studies showed that the higher expression of EGFR indicated higher tumor heterogeneity [17]. Thus, this relevance provided evidence that range has a correlation with EGFR mutation status. In our results, the sensitivity of range was 0.788, which was the highest among the three independent features. This indicated that the range had more advantages in diagnosing EGFR-positive mutation status. Another feature, skewness is used to describe a skewed distribution. The results of our study indicated that the EGFR-positive group had a lower skewness value than the EGFR-negative group. This finding was similar to that in previous studies on radiomics in genovariation of other tumors. For instance, in colorectal cancer, skewness was negatively correlated with KRAS mutation [18–20]. This might indicate that skewness was relatively universal in the genovariation of tumors, i.e., skewness might be a biomarker interrelated to the genetic phenotype. The ROC curve constructed with skewness had the highest specificity (0.909), which indicated that it was more reliable for diagnosis of EGFR negative expression. The previous research showed that range and skewness had a good predictive ability for EGFR mutation status (AUC 0.873, specificity 0.550, and sensitivity 0.900) in NSCLC [12]. Thus, not only the primary lung adenocarcinoma but also the bone metastases, range and skewness have a good judgment ability to EGFR mutation status. The last feature associated with EGFR mutation was quantile 0.975, which describes the central tendency of the voxel sample. When we constructed the ROC curve by combining the three features, the highest AUC value was obtained (AUC 0.783), which also had a higher sensitivity (0.708) and specificity (0.788). However, after combining every two features, we found that the AUC value, sensitivity and specificity of combination of range and skewness were the same as those of combination of the three features. This indicated that range and skewness could complement each other and their combination was sufficient to distinguish EGFR mutation positive or negative status, while quantile 0.975 was not particularly significant. Thus, quantile 0.975 may not be used for prediction of EGFR mutation status of the bone metastases in patients with primary lung adenocarcinoma.

According to our conclusion, histogram features play an important role in predicting EGFR mutation status in bone metastases. However, can we trust radiomics and be confident that radiomics can replace histology? Up to now, many studies have shown that radiomics can be used for histopathological classification, clinical stage, prediction of gene phenotype, efficacy evaluation and prognosis of tumors. For instance, radiomics could be used for predicting histology subtype in meningiomas, lymph node metastases in biliary tract cancer, and pathologic complete response after neoadjuvant chemoradiation therapy in rectal cancer, and their results were all medium and high predictive efficiency and higher sensitivity and specificity [21–25]. These studies indicate that the application prospects of radiomics are high potential. But radiomics still has some shortcomings, such as data non-standardization and single-center research. These lead to a lack of studies of large-scale randomized controlled trials in multi-center institutions. However, we can’t deny the potential of radiomics as an alternative to histology and ignore the clinical need for the radiomics. Because histologic sections and biopsies cannot solve the problem of tumor heterogeneity, which cannot reflect the information of whole tumor.

We acknowledge that our study has several limitations. Firstly, the sample size was small. Although our results were encouraging, experiments with a large sample size are needed to verify the results in the future. Secondly, the EGFR mutation status between primary pulmonary lesions and metastatic lesions was not completely consistent in one selected patient. In our study, it did not assess that whether CT-imaging based histogram analysis could be used to identify the EGFR mutation status of bone metastases in patients with the converse EGFR mutation status of primary lesions. We identified this problem. However, since this type of cases are relatively rare, we need to collect more such cases for further research.

## Conclusion

In conclusion, this study suggested a correlation between the CT imaging-based histogram features of bone metastases and their EGFR mutation status, and it also suggested that the CT histogram features were the biomarkers of EGFR. CT imaging-based histogram features might contribute to the diagnosis and prediction of EGFR mutation status of bone metastases in patients with primary lung adenocarcinoma. Although the treatment of metastases with EGFR-TKIs is still unclear, our study provides a new method for treatment selection and efficacy evaluation.

## Additional file

Additional file 1: **Figure S1.** The correlation heat map. For the color scale, dark blue indicates a positive correlation, while dark red indicates a negative correlation. The deeper the color, the stronger the relationship. “Group” indicates the EGFR status confirmed by gene detection. |R| > 0.9 was considered to indicate a strong relationship with each other. The color was lighter and all |R| values were no more than 0.7 between the three histogram features and the nine morphological features. **Table S1.** Results of DeLong’s test. (DOCX 232 kb)

## Data Availability

All authors have reviewed the final version of the paper and would like to take public responsibility for its content.

## Abbreviations

3D: Three-dimensional
AUC: Area under the curve
CI: Confidence interval
CT: Computed tomography
DICOM: Digital Imaging and Communications in Medicine
EGFR: Epidermal growth factor receptor
EGFR-TKI: Epidermal growth factor receptor tyrosine kinase inhibitor
FFPE: Formalin-fixed paraffin-embedded
MRMR: Minimum redundancy, maximum relevancy
NSCLC: Non-small cell lung cancer
PCR: Polymerase chain reaction
ROC: Receiver operating characteristic
ROI: Region of interests
