# Supplementary material for: CT imaging-based histogram features for prediction of EGFR mutation status of bone metastases in patients with primary lung adenocarcinoma
Source: Cancer Imaging. 2019 Jun 7;19:34. doi: 10.1186/s40644-019-0221-9 (PMC6556025; doi:10.1186/s40644-019-0221-9)
Supplement: Supplementary file 1 — Figure S1. The correlation heat map. For the color scale, dark blue indicates a positive correlation, while dark red indicates a negative correlation. The deeper the color, the stronger the relationship. “Group” indicates the EGFR status confirmed by gene detection. |R| > 0.9 was considered to indicate a strong relationship with each other. The color was lighter and all |R| values were no more than 0.7 between the three histogram features and the nine morphological features. Table S1. Results of DeLong’s test. (DOCX 232 kb) [file 40644_2019_221_MOESM1_ESM.docx]

Figure 1


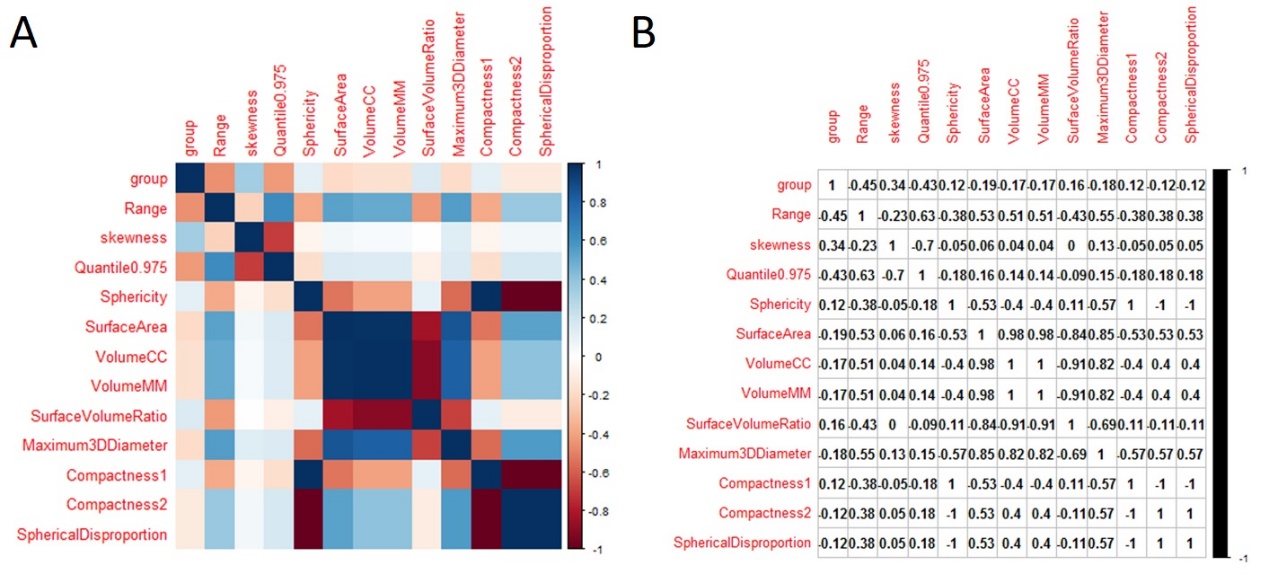


Fig.1 The correlation heat map. For the color scale, dark blue indicates a positive correlation, while dark red indicates a negative correlation. The deeper the color, the stronger the relationship. “Group” indicates the EGFR status confirmed by gene detection. |R|>0.9 was considered to indicate a strong relationship with each other. The color was lighter and all |R| values were no more than 0.7 between the three histogram features and the nine morphological features.

Table 1 Results of DeLong’s test.

| Feature | Range | Skewness | Quantile 0.975 | Combination  (R+S) | Combination  (R+Q) | Combination  (S+Q) | Combination  (R+S+Q) |
| --- | --- | --- | --- | --- | --- | --- | --- |
| Range | Z=0.0, p=1.0 | Z=0.7, p=0.5 | Z=0.2, p=0.8 | Z=-0.4, p=0.7 | Z=-0.1, p=0.9 | Z=0.2, p=0.9 | Z=-0.4, p=0.7 |
| Skewness | Z=0.7, p=0.5 | Z=0.0, p=1.0 | Z=0.8, p=0.4 | Z=-1.4, p=0.2 | Z=-0.9, p=0.3 | Z=-1.0, p=0.3 | Z=-1.0, p=0.2 |
| Quantile 0.975 | Z=0.2, p=0.8 | Z=0.8, p=0.4 | Z=0.0, p=1.0 | Z=-0.8, p=0.4 | Z=-0.6, p=0.5 | Z=-0.1, p=0.9 | Z=-0.8, p=0.4 |
| Combination  (R+S) | Z=-0.4, p=0.7 | Z=-1.4, p=0.2 | Z=-0.8, p=0.4 | Z=0.0, p=1.0 | Z=-0.9, p=0.3 | Z=0.7, p=0.4 | Z=0.0, p=1.0 |
| Combination  (R+Q) | Z=-0.1, p=0.9 | Z=-0.9, p=0.3 | Z=-0.6, p=0.5 | Z=-0.9, p=0.3 | Z=0.0, p=1.0 | Z=0.4, p=0.7 | Z=-0.5, p=0.6 |
| Combination  (S+Q) | Z=0.2, p=0.9 | Z=-1.0, p=0.3 | Z=-0.1, p=0.9 | Z=0.7, p=0.4 | Z=0.4, p=0.7 | Z=0.0, p=1.0 | Z=-0.8, p=0.4 |
| Combination  (R+S+Q) | Z=-0.4, p=0.7 | Z=-1.0, p=0.2 | Z=-0.8, p=0.4 | Z=0.0, p=1.0 | Z=-0.5, p=0.6 | Z=-0.8, p=0.4 | Z=0.0, p=1.0 |
